# Methylsulfonylmethane as a Bioactive Nutraceutical: Molecular Mechanisms, Clinical Evidence, Combination Therapy, Dosing, and Safety

**DOI:** 10.3390/ijms27208967

**Published:** 2026-10-09

**Authors:** Yonghyun Yoon, Jihyo Hwang, Jungyoun Kim, Jonghyeok Lee, Seungbeom Kim, Youngmo Kim, King Hei Stanley Lam

**Affiliations:** 1 Department of Orthopaedic Surgery, Hallym University Kangnam Sacred Heart Hospital, 1 Singil-ro, Yeongdeungpo-gu, Seoul 07441, Republic of Korea; 2 Incheon Terminal Orthopedic Surgery Clinic, Inha-ro 489beon-gil, Namdong-gu, Incheon 21574, Republic of Korea; 3 International Academy of Regenerative Medicine, Inha-ro 489beon-gil, Namdong-gu, Incheon 21574, Republic of Korea; 4 Bareun Neurosurgery Clinic, 39 Daenong-ro, Heungdeok-gu, Cheongju-si 28402, Republic of Korea; 5 Miso Pain Clinic, 1569 Bongyeong-ro, Yeongtong-gu, Suwon-si 16703, Republic of Korea; 6 Hongje Oneul Teun Teun Clinic, 442 Tongil-ro, Seodaemun-gu, Seoul 03629, Republic of Korea; 7 The Board of Clinical Research, International Academy of Musculoskeletal Medicine, Kowloon, Hong Kong SAR, China; 8 Faculty of Medicine, The University of Hong Kong, Hong Kong SAR, China; 9 Faculty of Medicine, The Chinese University of Hong Kong, New Territories, Hong Kong SAR, China

**Keywords:** methylsulfonylmethane, MSM, osteoarthritis, tendinopathy, nutraceuticals, NF-κB, NLRP3, combination therapy, safety, sulfur metabolism

## Abstract

Methylsulfonylmethane (MSM; dimethyl sulfone) is an organosulfur nutraceutical used primarily for joint symptoms, although its molecular actions and clinical applicability beyond osteoarthritis remain uncertain. This narrative review integrates pharmacological, preclinical, and human evidence on MSM, with emphasis on musculoskeletal disorders, multi-ingredient formulations, dosing, safety, toxicology, and clinical monitoring. Experimental studies implicate NF-κB and NLRP3 signaling, redox regulation, sulfur metabolism, and AMPK/mTOR/ULK1-related autophagy; however, most pathway-level evidence remains preclinical. Human clinical evidence is comparatively most developed for knee osteoarthritis, although it remains limited to small, short-term randomized trials reporting modest symptomatic improvement rather than established structural or disease-modifying benefit. Recent evidence syntheses also identify signals for glucosamine–MSM and glucosamine–chondroitin–MSM combinations, although certainty is limited and the independent contribution of MSM cannot be determined. Tendon studies largely evaluate multi-ingredient products, preventing attribution of benefit to MSM alone. Evidence in cardiometabolic disease, gastrointestinal health, exercise recovery, and toxicant exposure is preliminary. MSM is not a direct metal chelator and should not replace established chelation therapy. Short-term studies have used approximately 1–6 g/day with generally mild gastrointestinal adverse effects, whereas long-term safety, formal drug-interaction data, and clinically validated biomarker-guided dosing remain undefined. MSM should therefore be considered an adjunct with indication-dependent evidence rather than a broadly applicable anti-inflammatory or detoxification supplement.

## 1. Introduction

Methylsulfonylmethane (MSM; dimethyl sulfone) is a naturally occurring organosulfur compound present at low concentrations in foods and human biological fluids. It is widely marketed for joint discomfort and osteoarthritis (OA), either alone or in combination with other nutraceuticals [1,2,3]. Despite widespread use, the human evidence base remains relatively small, short-term, and heterogeneous.

Interpretation is further complicated by the frequent use of MSM in multi-ingredient products. The clinically relevant questions are therefore not limited to monotherapy efficacy, but also include the incremental contribution of MSM within combination regimens, dose and treatment duration, safety in patients with comorbidities or polypharmacy, and the use of objective outcomes to determine whether supplementation should be continued.

MSM is also promoted for cardiometabolic, gastrointestinal, and toxicant-related indications. These claims require a clear distinction between molecular plausibility and clinical efficacy. In particular, sulfur content alone does not confer metal-chelating activity, and antioxidant, sulfur-metabolic, or permeability effects should not be interpreted as evidence that MSM removes toxic metals from the human body.

Our group recently reported rapid clinical and radiographic improvement in a patient with large multilobulated shoulder calcific tendinitis treated with ultrasound-guided prolotherapy and a multimodal regimen that included MSM, L-arginine, vitamin C, vitamin D, and magnesium [4]. Because the intervention was multimodal and spontaneous resorption may occur, the report is hypothesis-generating and does not establish an MSM-specific effect.

This narrative review integrates the molecular pharmacology of MSM with human clinical evidence; evaluates monotherapy and combination use in musculoskeletal disorders; distinguishes clinical findings from preclinical signals in cardiometabolic, gastrointestinal, and toxicant-related settings; and summarizes dosing, safety, drug-interaction, and monitoring considerations. Previous nutraceutical reviews have discussed MSM within broader osteoarthritis frameworks [5,6], whereas the present review focuses specifically on MSM and the gap between its broad mechanistic literature and its narrower clinical evidence base.

## 2. Literature Identification and Narrative Evidence Synthesis

This narrative review was informed by an illustrative, targeted literature search because the evidence spans randomized trials, observational and case-level human evidence, pharmacokinetic studies, animal experiments, and in vitro mechanistic work.

PubMed/MEDLINE, ScienceDirect, and Google Scholar were searched from database inception through 26 September 2026. Search concepts combined MSM synonyms (“methylsulfonylmethane,” “methyl sulfonyl methane,” “methylsulphonylmethane,” “dimethyl sulfone,” “dimethyl sulphone,” and “MSM” when coupled with supplement-related terms) with indication, mechanism, formulation, safety, and interaction terms. The full search framework and representative source-specific queries are provided in Supplementary Table S1. Reference lists of relevant reviews and primary studies were screened for additional eligible reports.

Eligible evidence included peer-reviewed human randomized or controlled studies, observational studies, case-level reports, pharmacokinetic or biomarker studies, safety or interaction reports, and systematic reviews or meta-analyses that specifically evaluated MSM monotherapy or an explicitly identified MSM-containing formulation. Animal and in vitro studies were retained only for mechanistic questions or clinical domains with sparse or absent human evidence. Non-peer-reviewed promotional material, reports in which MSM exposure could not be established, and nonhuman studies used as direct evidence of clinical efficacy were excluded. When multiple reports addressed the same study or formulation, the most complete source was prioritized and companion information was used only to clarify methods or outcomes.

Potentially relevant publications were screened for topical relevance at the title/abstract level and assessed in full text when eligibility or interpretation was uncertain. The included evidence and its interpretation were reviewed by the authors during manuscript verification. Evidence was considered descriptively in the following hierarchy: systematic reviews or meta-analyses of controlled human studies; randomized controlled trials; nonrandomized or observational human studies; case-level evidence; animal studies; and in vitro studies. This hierarchy guided narrative emphasis and was not a formal certainty-of-evidence grading system.

**Figure 1:**
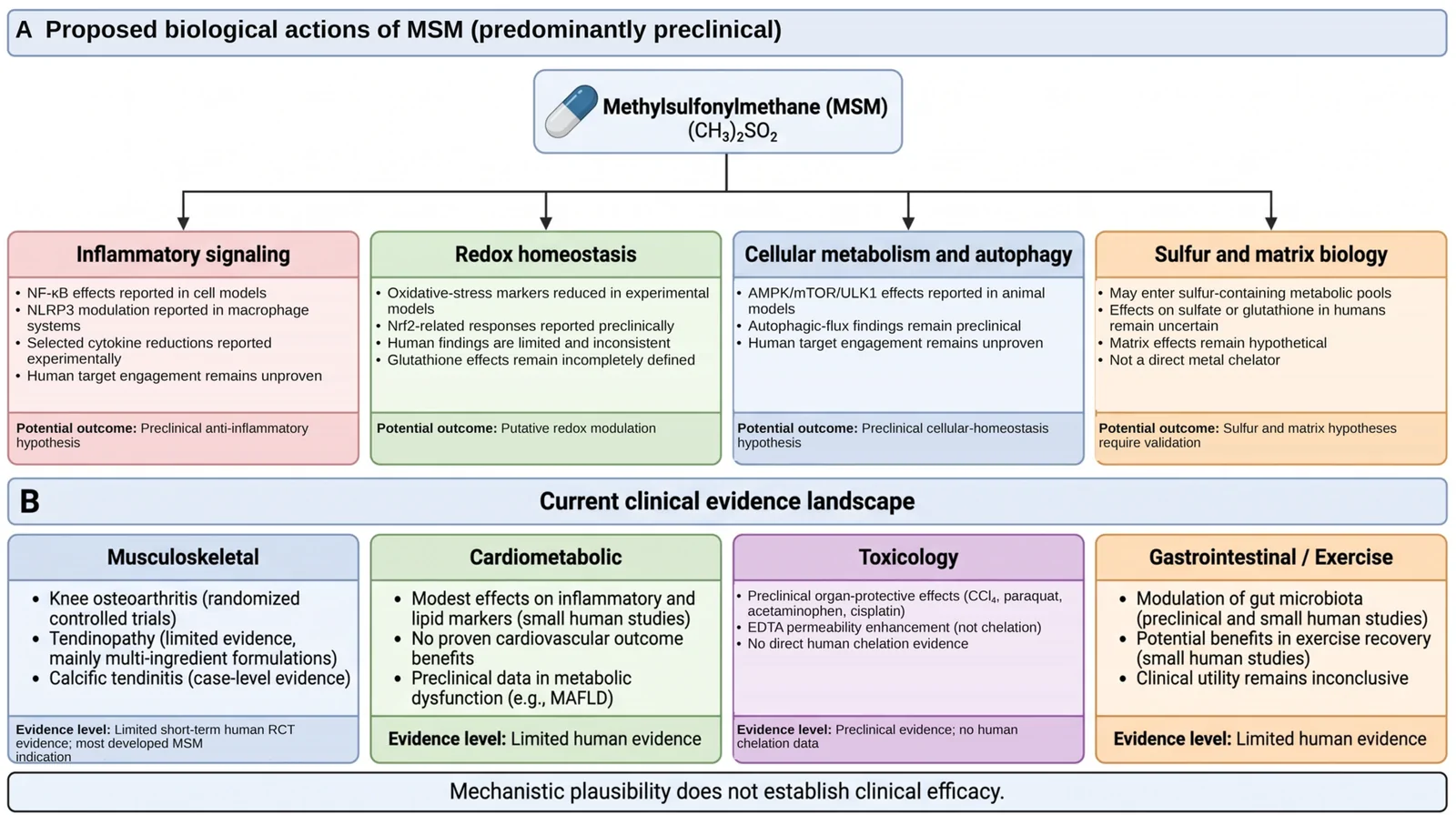
Proposed biological actions of methylsulfonylmethane (MSM) and the current clinical evidence landscape. Panel A summarizes reported mechanisms, most of which derive from in vitro or animal studies; the diagram does not imply human target engagement at clinically used oral doses. Panel B uses descriptive, domain-specific evidence categories rather than a formal GRADE assessment. COX-2, cyclooxygenase-2; CRP, C-reactive protein; EDTA, ethylenediaminetetraacetic acid; HDL, high-density lipoprotein; IL, interleukin; iNOS, inducible nitric oxide synthase; MAFLD, metabolic-associated fatty liver disease; mTOR, mechanistic target of rapamycin; NF-κB, nuclear factor kappa B; NLRP3, NOD-like receptor family pyrin domain-containing 3; Nrf2, nuclear factor erythroid 2-related factor 2; ROS, reactive oxygen species; TNF-α, tumor necrosis factor alpha; ULK1, unc-51-like kinase 1. Created in BioRender. Yoon, M. (2026) https://BioRender.com/vkn40qy (accessed on 1 September 2026).

Because this was a narrative review, no formal risk-of-bias instrument or de novo meta-analysis was applied, no PRISMA flow diagram was constructed, and search-result counts were not retrospectively inferred. The review should therefore not be interpreted as an exhaustive systematic review. Generative AI tools were used to support manuscript organization, language refinement, and preliminary reference cross-checking. The authors independently verified all scientific claims, numerical values, and cited sources against the underlying publications. The conceptual structure and evidence classifications in Figure 1 were defined by the authors, and the final artwork was created by the authors in BioRender; generative AI did not independently select, grade, or generate the figure’s scientific content.

## 3. Molecular Pharmacology and Mechanisms of Action of MSM

MSM is a small, water-soluble sulfone that is systemically bioavailable after oral administration. In rats, radiolabeled MSM is rapidly absorbed, widely distributed, and predominantly eliminated in urine [7,8]. These animal data establish absorption and disposition but do not define a human therapeutic half-life or dosing interval. In humans, dimethyl sulfone has been detected in plasma, cerebrospinal fluid, urine, and brain tissue [9,10,11], confirming systemic exposure while leaving dose–exposure relationships incompletely characterized. Figure 1 summarizes the proposed biological actions and the relative maturity of clinical evidence.

Experimental studies most consistently implicate inflammatory and redox pathways, including NF-κB-related signaling, NLRP3 inflammasome activation, reactive oxygen species, and antioxidant responses [1,12]. Additional preclinical work suggests effects on cellular metabolism, autophagy, and sulfur-related pathways. These proposed actions are pathway-level hypotheses; they do not establish human target engagement at clinically used oral doses or clinical efficacy.

MSM is part of the broader sulfur metabolome, but its quantitative contribution to human sulfur flux is not well defined [13]. Methionine, cysteine, glutathione, sulfite, thiosulfate, and sulfate are connected through distinct metabolic pathways. MSM lacks the free thiol groups characteristic of major dithiol chelators; sulfur metabolism, glutathione homeostasis, and direct metal chelation should therefore be treated as separate concepts.

### 3.1. NF-κB Signaling and Pro-Inflammatory Cytokines

In LPS-stimulated RAW264.7 macrophages, MSM reduced nitric oxide and prostaglandin E2 production, downregulated iNOS and COX-2, decreased IL-6 and TNF-α release, and inhibited NF-κB p65 nuclear translocation [14]. MSM also suppressed RANKL-induced osteoclastogenesis through NF-κB and STAT3 signaling in bone-marrow-derived macrophages [15]. In a human THP-1 monocyte cell model, MSM attenuated high-glucose-induced TLR2/TLR4 expression, ROS-related NF-κB signaling, and expression of IL-1β, IL-6, and TNF-α [16]. These studies support pathway-level activity but do not establish equivalent target engagement in patients.

### 3.2. NLRP3 Inflammasome Modulation

MSM inhibited NLRP3 inflammasome activation in mouse and human macrophage systems, with reductions in NLRP3-related signaling, pro-inflammatory cytokine transcription, and mitochondrial reactive oxygen species [12]. The reported effect was selective for NLRP3 relative to NLRC4 and AIM2 inflammasomes. Whether clinically used oral doses suppress NLRP3 activity in humans has not been demonstrated.

### 3.3. Oxidative Stress and Nrf2-Related Responses

Preclinical models report reductions in oxidative-stress markers and, in some settings, Nrf2-related responses [1,17,18]. Direct evidence of Nrf2 activation in humans receiving MSM is lacking. Limited human target-engagement data are available: in a small randomized exercise study, 10 days of MSM at 50 mg/kg/day attenuated selected oxidative-stress markers and increased the GSH/GSSG ratio [19]. Other pilot studies reported variable effects on antioxidant capacity, cytokine responses, or perceived recovery [20,21,22]. These findings are compatible with possible redox modulation but do not establish a general clinical antioxidant indication.

### 3.4. AMPK/mTOR/ULK1 Signaling and Autophagy

In a high-fat-diet mouse model of metabolic-associated fatty liver disease, MSM restored autophagic flux through the AMPK/mTOR/ULK1 pathway and improved insulin sensitivity, hepatic steatosis, and biochemical markers [23]. This pathway remains preclinical and should not be extrapolated to treatment of human metabolic disease.

### 3.5. Sulfur Metabolism and Chelation Chemistry

The sulfur-related biology of MSM is less clearly defined than its inflammatory and redox effects. Although MSM may enter sulfur-containing metabolic pools, direct human evidence that supplementation materially increases sulfate availability or glutathione synthesis is limited. Metal chelation requires donor atoms with available electron density arranged so that a metal ion can be coordinated with sufficient affinity. In MSM, sulfur is present in the oxidized sulfone state, bonded to two oxygen atoms and two methyl groups, and the molecule contains no free thiol (–SH) donor groups. Accordingly, sulfur content alone does not confer the metal-binding chemistry of dithiol chelators such as DMSA or DMPS [24,25]. Sulfur metabolism, glutathione homeostasis, permeability enhancement, and direct metal chelation should therefore be treated as distinct concepts.

### 3.6. Mechanistic Rationale for Combination Formulations

MSM-containing products often combine compounds with distinct biological roles. L-arginine provides substrate for nitric oxide synthesis and repair signaling [26,27,28], collagen and lysine provide matrix-related amino-acid substrates, vitamin C is required for collagen hydroxylation [29], and glucosamine/chondroitin are used as glycosaminoglycan-oriented joint supplements. This rationale is mechanistically coherent but does not demonstrate pharmacological synergy; the clinical combination evidence is reviewed in Section 4.3.

## 4. Clinical Evidence in Musculoskeletal Disorders

### 4.1. Osteoarthritis

Human clinical evidence is comparatively most developed for mild-to-moderate knee OA, but it remains limited to small, short-term trials. In a pilot randomized, double-blind, placebo-controlled trial of 50 adults, Kim et al. administered MSM 3 g twice daily (6 g/day) for 12 weeks. Between-group improvements were statistically significant for WOMAC pain (*p* = 0.041) and physical-function impairment (*p* = 0.045), but the trial was small and did not establish clinical importance or durability [2]. Debbi et al. randomized 49 participants with radiographically confirmed knee OA to MSM 1.125 g three times daily (3.375 g/day) or placebo for 12 weeks. Adjusted between-group differences favored MSM for WOMAC physical function (14.6 mm, 95% CI 4.3–25.0; *p* = 0.04) and WOMAC total score (15.0 mm, 95% CI 5.1–24.9; *p* = 0.03), whereas WOMAC pain and stiffness did not differ significantly (both *p* = 0.08); the authors judged the improvements small and of uncertain clinical significance [30].

In 88 healthy Japanese adults with mild knee pain rather than established radiographic OA, MSM 2 g/day for 12 weeks produced a between-group difference in the JKOM total score at week 12 (*p* = 0.046) and in the JKOM health-condition component (*p* = 0.032) [31]. These findings should not be extrapolated directly to disease modification in established OA. Overall evidence remains limited by small samples, short follow-up, heterogeneous populations and doses, incomplete reporting of effect magnitudes and adverse events, and no convincing structural endpoints [32]. A 2025 network meta-analysis of dietary supplements ranked MSM highly for WOMAC pain, but the certainty of evidence was low and larger trials were recommended [33]. MSM 3 g/day also did not prevent knee symptoms in 180 military initial-entry trainees [34]. The current evidence therefore supports, at most, a time-limited symptomatic adjunct in selected patients rather than preventive, structural, or disease-modifying use.

### 4.2. MSM with Glucosamine and Chondroitin

In a 12-week, four-arm RCT of 118 patients with knee OA, MSM 500 mg three times daily, glucosamine 500 mg three times daily, their combination, and placebo were compared. The MSM monotherapy group showed a 33% reduction in VAS pain (*p* < 0.001), while the combination produced the largest and most rapid improvements in pain and swelling indices [3]. Because the study did not demonstrate a formal component-by-component interaction, the result supports a possible additive clinical signal but not pharmacological synergy.

Recent evidence synthesis provides additional context. A 2024 network meta-analysis of 30 RCTs reported a clinically important overall-pain effect for glucosamine + chondroitin sulfate + MSM versus placebo, although certainty was low; glucosamine + MSM also showed a large effect estimate with uncertainty around the minimum clinically important difference [35]. These findings justify further study of complete regimens but do not isolate the incremental contribution of MSM or establish synergy among components.

### 4.3. MSM with Amino Acids, Collagen, and Tendon-Directed Formulations

Several proprietary tendon formulations combine MSM with amino acids, collagen-derived substrates, and other bioactives. Tendisulfur Forte contains MSM, hydrolyzed type I and II collagen, L-arginine, L-lysine, vitamin C, chondroitin sulfate, glucosamine, Curcuma longa, Boswellia serrata, and myrrh [36]. Tenosan contains arginine L-alpha-ketoglutarate, MSM, hydrolyzed type I collagen, and bromelain [37]. L-arginine has a plausible repair rationale through nitric-oxide biology [26,27,28], whereas collagen-derived substrates and vitamin C relate to extracellular-matrix synthesis [29]. These are formulation-level studies and should not be interpreted as MSM-monotherapy evidence; their clinical effects cannot be assigned to MSM.

#### Co-Administered Nutrients and Bioactives in MSM-Containing Regimens

Co-administration is a recurrent feature of the musculoskeletal literature. MSM has been studied with glucosamine and chondroitin, L-arginine, L-lysine, hydrolyzed collagen, vitamin C, bromelain, and botanical compounds [36,37,38]. In our calcific tendinitis case, MSM was co-administered with L-arginine, vitamin C, vitamin D, and magnesium [4]. Table 1 summarizes the reported co-ingredients, their complementary biological rationale, and the principal interpretive limitation: the absence of component-isolating factorial trials.

Vitamin C has a direct biochemical role in collagen hydroxylation and tissue repair [29,39]. Vitamin D contributes to musculoskeletal and calcium-phosphate physiology, whereas magnesium serves as an enzymatic cofactor with neuromuscular, vascular, and metabolic functions [40,41]. These independent biological roles justify mechanistic discussion but do not demonstrate that any of these nutrients potentiates the effect of MSM.

In a prospective randomized study of 90 patients undergoing arthroscopic repair of large posterosuperior rotator cuff tears, a three-month Tenosan regimen was associated with lower shoulder pain at six months and a modest improvement in MRI repair integrity, while objective functional outcomes did not significantly differ between groups [37]. This pattern suggests a potential effect on early pain and structural healing without proven long-term functional superiority.

A non-randomized comparative study evaluated extracorporeal shockwave therapy (ESWT) alone versus ESWT plus Tendisulfur Forte in 90 patients with rotator cuff tendinopathy, lateral epicondylitis, or Achilles tendinopathy [36]. At 60 days, the combination group demonstrated better UCLA shoulder scores, Mayo elbow scores, VISA-A improvement, and lower pain scores, together with reduced NSAID consumption. The supplement was administered twice daily for one month and once daily for a second month. The investigators advised administration on an empty stomach to increase presumed bioavailability; however, this protocol does not establish that fasting improves MSM pharmacokinetics or clinical efficacy.

A separate randomized placebo-controlled study of Tendisulfur after arthroscopic supraspinatus repair reported lower early overall and night pain but no sustained difference in later Constant-Murley or Simple Shoulder Test scores [38]. Systematic and scoping reviews of nutrition in tendinopathy similarly describe promising signals for supplement-based adjuncts but emphasize the small, heterogeneous evidence base and the inability to identify an optimal formulation [42,43].

### 4.4. Calcific Tendinitis and Other Tendinopathies

Calcific tendinitis of the rotator cuff is managed with activity modification, rehabilitation, ultrasound-guided lavage or barbotage, corticosteroid injection, extracorporeal shockwave therapy (ESWT), and selected surgical procedures; contemporary tendinopathy reviews emphasize the heterogeneity of treatment response and the need for condition-specific strategies [44]. No controlled trial has evaluated MSM monotherapy in calcific tendinitis. Our published case involved a 49-year-old man with severe pain (VAS 10/10) and an approximately 3-cm multilobulated deposit who received ultrasound-guided 10% dextrose prolotherapy together with hydration, physical therapy, activity modification, vitamin C 10 g/day, vitamin D 5000 IU/day, magnesium 500 mg/day, MSM 2000 mg/day, and L-arginine 2000 mg/day [4]. At two weeks, pain had decreased to 2/10, active range of motion was full with mild terminal pain, and radiographs showed near-complete resorption.

The rapid course is notable but cannot be attributed to MSM or any individual co-intervention because calcific tendinitis may enter a spontaneous resorptive phase and all treatments were initiated concurrently. The case therefore supports hypothesis generation and prospective study design, not an efficacy claim.

### 4.5. Exercise-Induced Muscle Damage and Recovery

Exercise studies provide a human model of oxidative and inflammatory stress, but results are inconsistent. MSM 50 mg/kg/day for 10 days reduced selected post-exercise oxidative-stress markers in 18 untrained men [19]. A small dose-comparison pilot suggested possible effects on soreness, fatigue, and antioxidant capacity [21], whereas a placebo-controlled half-marathon trial found no significant treatment effect on oxidative-stress or muscle-damage biomarkers; pain differences were clinically suggestive but not statistically significant [20]. MSM has also altered ex vivo cytokine responses in a small study [22]. The discrepant findings may reflect heterogeneity in training status, exercise stimulus (brief exhaustive exercise versus endurance racing), dose and duration, timing of post-exercise sampling, selected biomarkers, and small sample sizes. The studies therefore do not demonstrate a reproducible biomarker or clinical effect and do not establish a performance-enhancing or injury-preventive indication. Key clinical studies are summarized in Table 2.

## 5. Beyond Musculoskeletal Disease

### 5.1. Heavy-Metal and Xenobiotic Exposure: Chelation, Permeability, and Oxidative Injury

MSM should not be described as a clinically validated heavy-metal “detoxification” agent. Established chelation therapy for arsenic, mercury, or lead intoxication uses agents with defined metal-binding chemistry, including DMSA, DMPS, BAL, or calcium disodium EDTA according to the toxicant and clinical setting [24,25]. MSM is a sulfone, lacks free thiol donor groups, and should not be considered chemically interchangeable with dithiol chelators.

Sodium thiosulfate is a distinct sulfur-containing drug used in several toxicological and metabolic settings [45]. Its pharmacology is not interchangeable with MSM; structural sulfur content alone is insufficient to infer equivalent detoxification activity.

The most directly relevant chelation-related MSM study evaluated MSM as a permeability enhancer rather than as a chelator. In rats, topical MSM facilitated ocular penetration of EDTA, whereas EDTA alone penetrated poorly [46]. This supports a delivery or permeability hypothesis, not systemic metal chelation.

Animal studies also report attenuation of carbon-tetrachloride-induced hepatic injury and paraquat-induced lung and liver injury [17,18]. Additional preclinical studies found that MSM pretreatment attenuated acetaminophen-induced hepatotoxicity in rats and mitigated early cisplatin-induced testicular oxidative dysfunction in rats, with effects centered on glutathione/antioxidant defenses and redox homeostasis [47,48]. These findings are more accurately described as antioxidant or organ-protective effects rather than detoxification or chelation. No controlled human study has shown that oral MSM alone lowers blood or tissue metal burden or increases clinically meaningful metal elimination.

Future toxicology studies should distinguish direct chelation from redox modulation, sulfur metabolism, and permeability effects and should use metal-specific toxicokinetic and clinical endpoints.

### 5.2. Cardiometabolic and Atherosclerotic Contexts

Human cardiometabolic evidence is limited to surrogate outcomes. In a 16-week randomized trial of overweight or obese adults, MSM 3 g/day increased HDL cholesterol and reduced C-reactive protein at selected time points, without significant changes in LDL cholesterol, triglycerides, glucose, or insulin [49]. Preclinical studies report effects in pulmonary hypertension and metabolic-associated fatty liver disease, including oxidative-stress modulation and AMPK/mTOR/ULK1-related autophagy [23,50].

No human study has demonstrated improvement in carotid intima-media thickness, coronary artery calcium, cardiovascular events, or other direct atherosclerotic outcomes. The available findings should therefore be regarded as preliminary biomarker or mechanistic signals.

### 5.3. Gastrointestinal and Microbiome-Related Considerations

Direct human evidence for gastrointestinal indications is sparse. Dimethyl sulfone participates in microbial–mammalian co-metabolism, and its concentration may reflect diet, microbial metabolism, and endogenous methanethiol pathways [9,51]. This metabolic association does not demonstrate therapeutic efficacy for inflammatory bowel disease, irritable bowel syndrome, intestinal permeability, or nonspecific “gut health.”

## 6. Comorbidity, Polypharmacy, and Drug-Interaction Considerations

No validated MSM dose-adjustment algorithms exist for chronic kidney disease, chronic liver disease, diabetes, atherosclerotic disease, or frailty. Evidence is insufficient to infer safety in these populations from small short-term trials conducted in healthier participants. Renal and hepatic function, concomitant medications, and the total supplement burden should therefore be considered when MSM is used clinically.

### 6.1. Anticoagulant and Antiplatelet Therapy

Warfarin requires particular attention because MSM-containing joint products often also contain glucosamine and chondroitin. The documented interaction signal is associated primarily with glucosamine and/or chondroitin: case and pharmacovigilance reports describe increases in the international normalized ratio (INR) and bleeding or bruising after these products were started or their dose was increased [52]. The direction of concern is potentiation of anticoagulation, not a procoagulant effect. In the databases and reference lists reviewed, we did not identify a published case report or pharmacovigilance report in which a clinically significant warfarin interaction was clearly attributable to MSM monotherapy. This absence of identified evidence should not be interpreted as proof that an interaction is impossible.

Adverse events after a multi-ingredient product should therefore not automatically be attributed to MSM. In patients taking warfarin, the complete ingredient list should be reviewed and closer INR surveillance is reasonable when glucosamine/chondroitin-containing products are started, stopped, or substantially changed.

### 6.2. Other Drug-Interaction and Polypharmacy Gaps

No controlled human interaction studies have evaluated MSM with direct oral anticoagulants, antiplatelet agents, glucose-lowering drugs, antihypertensive agents, or medicines that depend heavily on renal or hepatic clearance. In the absence of such data, interaction risk cannot be inferred from short-term monotherapy trials in healthier participants. Clinical use in polypharmacy should include review of the full formulation, renal and hepatic function, temporal relationships between supplement changes and adverse events, and discontinuation when a plausible interaction is suspected.

## 7. Dose, Administration, and Duration

Human studies have used approximately 1–6 g/day, commonly divided into two or three doses, with most interventions lasting 4–16 weeks [2,19,20,21,22,30,31]. The evidence does not identify a universal minimum effective dose, dose–response plateau, or maintenance regimen. Lower-to-mid gram doses are more frequently represented across indications, whereas higher doses and long-term exposure are less well characterized.

Where MSM is used clinically, dose escalation should be conservative and linked to a predefined symptomatic or functional target rather than to an assumption that higher intake is more effective.

### Fasting Versus Fed Administration

Some tendon protocols have administered MSM-containing formulations on an empty stomach; the Tendisulfur Forte study used this instruction to increase presumed bioavailability [36]. No controlled fed-versus-fasted human pharmacokinetic study demonstrates superior MSM exposure or clinical efficacy with fasting administration. Administration should therefore be guided primarily by tolerability until comparative pharmacokinetic data are available.

## 8. Safety, Tolerability, and Toxicity

Short-term studies generally describe MSM as well tolerated across approximately 1–6 g/day [2,20,21,22,34,49]. Reported adverse effects are usually mild and gastrointestinal, including nausea, dyspepsia, bloating, and diarrhea, with occasional headache or nonspecific symptoms. Safety reporting has been inconsistent, and the absence of serious events in small short-duration studies does not establish long-term safety. These observations apply only to the populations, formulations, doses, and exposure windows actually studied.

Important evidence gaps include prolonged continuous use, pregnancy and lactation, clinically significant renal or hepatic impairment, frailty, and formal drug-interaction studies. No robust human evidence defines cumulative toxicity, withdrawal effects, or the safety of multi-year exposure. Safety conclusions should therefore remain restricted to the doses, durations, formulations, and populations actually studied, with active review of adverse effects and co-administered supplements or medicines.

## 9. Biomarkers and Clinical Monitoring: Current Limits

No validated laboratory biomarker currently identifies an MSM-deficient state, predicts response, or defines an optimal dose. Clinical monitoring should therefore be anchored primarily to the target disorder rather than to nonspecific supplement panels.

### 9.1. Measurement of Dimethyl Sulfone

Dimethyl sulfone can be quantified in urine by gas chromatography–mass spectrometry and detected in plasma, cerebrospinal fluid, and other tissues [9,11]. Measurable concentration does not define deficiency, therapeutic adequacy, or toxicity. Concentrations may vary with diet, endogenous metabolism, microbial metabolism, and supplementation; the onion-derived dimethyl-sulfone biomarker study, for example, was conducted in rats rather than humans [53].

Commercial organic-acid or metabolomic panels may report sulfur-related metabolites, but no validated dimethyl-sulfone target exists for selecting or titrating an MSM dose. Organic-acid testing should therefore be considered exploratory, not a validated prescribing tool.

### 9.2. Indication-Specific Outcome Monitoring

Outcome monitoring should be indication-specific: pain plus WOMAC, KOOS, or JKOM for knee OA; VISA-A for Achilles tendinopathy; validated shoulder scores for rotator cuff disorders; and validated metal-specific blood or urine testing when toxic-metal exposure is suspected. Cardiometabolic investigations should use conventional disease-specific endpoints rather than MSM-specific surrogate panels.

## 10. Practical Interpretation of the Current Evidence

The following points are intended as an interpretive framework rather than a clinical guideline or validated treatment algorithm. They summarize how the available evidence can be translated cautiously into patient selection, time-limited use, outcome assessment, and discontinuation decisions.

1. Define the indication and baseline outcome. Establish the diagnosis, symptom duration, prior treatment, and at least one validated baseline outcome. Avoid using MSM for nonspecific “inflammation” or “detoxification” without a defined clinical target.

2. Match the proposed use to the evidence and review co-exposures. Distinguish evidence for MSM monotherapy from evidence for multi-ingredient products, and review comorbidities, medications, anticoagulants, and concurrent supplements.

3. Use a time-limited, evidence-informed dosing strategy. Human studies span approximately 1–6 g/day, but no dose–response plateau has been established. Escalation should be driven by tolerability and a predefined clinical target.

4. Reassess the same outcome and adverse effects. For joint or tendon symptoms, reassess the prespecified validated measure after a reasonable trial period, commonly 8–12 weeks in the available studies, while actively assessing gastrointestinal and other adverse effects.

5. Continue only when benefit is demonstrable. If clinically meaningful improvement is absent, discontinue MSM or reconsider the treatment strategy rather than continuing solely because the product is perceived as natural or safe. Table 3 summarizes the proposed domain-specific evidence and monitoring approach.

## 11. Evidence Gaps and Priorities for Future Research

Priority research questions are: (1) factorial or component-isolating trials to determine the incremental contribution of MSM within glucosamine/chondroitin and tendon-directed formulations; (2) prospective controlled studies in calcific and non-calcific tendinopathy that separate local procedures from nutritional adjuncts; (3) dose-ranging and human pharmacokinetic studies, including fed-versus-fasted comparisons; and (4) biomarker studies to determine whether plasma or urinary dimethyl sulfone has any role in adherence, exposure, pharmacokinetics, or response. Current data do not support deficiency thresholds or organic-acid-test-guided dosing.

Additional priorities are: (5) toxicology studies that distinguish chelation from redox or permeability effects and measure metal-specific body burden or excretion; (6) dedicated safety studies in patients with renal or hepatic impairment and polypharmacy; and (7) longer-term safety, pregnancy/lactation, and formal drug-interaction studies.

## 12. Conclusions

MSM has a biologically plausible but clinically uneven evidence base. Human evidence is comparatively most developed for short-term symptomatic outcomes in knee OA, but the trials remain small, heterogeneous, and insufficient to establish structural or disease-modifying benefit. Evidence syntheses suggest additional signals for glucosamine–MSM and glucosamine–chondroitin–MSM combinations [33,35], but these do not isolate the independent contribution of MSM or prove synergy. Tendon studies are primarily formulation-level and cannot establish an MSM-specific effect. Cardiometabolic, gastrointestinal, exercise, toxicant-related, and connective-tissue applications remain preliminary, inconsistent, or preclinical.

The molecular literature is broader than the clinical evidence and should be interpreted accordingly. MSM is not a direct metal chelator, validated biomarker-guided dosing is unavailable, formal interaction data are sparse, and long-term safety remains incompletely characterized. Where MSM is used, a time-limited adjunctive approach with a defined indication, a validated baseline outcome, explicit reassessment, and discontinuation in the absence of measurable benefit is more defensible than unrestricted long-term supplementation.

## Figures and Tables

**Table 1:** Co-administered nutrients and bioactives reported in MSM-containing musculoskeletal regimens.

Co-Administered Component	MSM-Containing Clinical Context	Complementary Rationale	Interpretive Limitation
Glucosamine ± chondroitin	Knee OA; glucosamine + MSM RCT [3]; G + CS + MSM in network meta-analysis [35]	Cartilage/ECM and glycosaminoglycan-oriented support	The incremental contribution of MSM remains incompletely isolated; reported warfarin interaction signals relate primarily to glucosamine and/or chondroitin.
L-arginine/L-lysine	Tendisulfur/Tenosan; calcific tendinitis case [4,36,37]	NO-related repair signaling; amino-acid substrate for connective tissue [26,27,28]	No factorial trial demonstrates synergy with MSM.
Hydrolyzed collagen	Tendisulfur/Tenosan [36,37]	Provides collagen-derived amino-acid substrate for ECM turnover	Clinical effects belong to the complete formulation.
Vitamin C	Tendon formulations; calcific tendinitis case [4,36,39]	Collagen hydroxylation, redox regulation, tissue repair [29,39]	No evidence that vitamin C specifically potentiates MSM.
Vitamin D + magnesium	Calcific tendinitis case [4,40,41]	Musculoskeletal/calcium-phosphate homeostasis (vitamin D); enzymatic, neuromuscular and vascular/metabolic cofactor functions (magnesium)	Case-level co-administration only; no MSM-specific interaction or calcific-resorption effect established.
Bromelain/Curcuma/Boswellia/myrrh	Tenosan and Tendisulfur formulations [36,37,38]	Anti-inflammatory and analgesic rationale	Multi-ingredient confounding prevents component attribution.

Abbreviations: CS, chondroitin sulfate; ECM, extracellular matrix; G, glucosamine; MSM, methylsulfonylmethane; NO, nitric oxide; OA, osteoarthritis.

**Table 2:** Selected human clinical studies of MSM monotherapy, MSM-containing combinations, and multimodal regimens.

Study; Population and Design	MSM Exposure	Comparator and Follow-Up	Main Quantitative Result	MSM Attribution	Key Limitation/Clinical Interpretation
Usha & Naidu, 2004 [3] Knee OA; four-arm double-blind RCT; n = 118	MSM 500 mg three times daily; glucosamine 500 mg three times daily; combination; 12 weeks	Placebo; 12 weeks	MSM arm: 33% VAS pain reduction (*p* < 0.001); combination produced the largest and fastest symptom improvement	Direct MSM arm plus combination arm	No formal interaction test; synergy not established; outcome reporting limits clinical interpretation
Kim et al., 2006 [2] Knee OA; pilot double-blind RCT; n = 50	MSM 6 g/day; 12 weeks	Placebo; 12 weeks	Between-group WOMAC pain *p* = 0.041; physical function *p* = 0.045	MSM monotherapy	Pilot sample; effect magnitude, clinical importance, and durability remain uncertain
Debbi et al., 2011 [30] Radiographic knee OA; double-blind RCT; n = 49	MSM 3.375 g/day; 12 weeks	Placebo; 12 weeks	Physical function: 14.6 mm (95% CI 4.3–25.0; *p* = 0.04); total: 15.0 mm (5.1–24.9; *p* = 0.03); pain/stiffness *p* = 0.08	MSM monotherapy	Small study; adjusted analysis; authors considered improvements small and of uncertain clinical significance
Toguchi et al., 2023 [31] Healthy adults with mild knee pain; double-blind RCT; n = 88	MSM 2 g/day; 12 weeks	Placebo; 12 weeks	JKOM total *p* = 0.046; JKOM health-condition component *p* = 0.032 at week 12	MSM monotherapy	Population did not require established radiographic OA; no structural endpoint
Tennent et al., 2017 [34] Military trainees; preventive RCT; n = 180	MSM 3 g/day; 8 weeks	Placebo; 8 weeks	No significant improvement in KOOS or POMS	MSM monotherapy	Negative prevention trial; not a treatment study in symptomatic OA
Nakhostin-Roohi et al., 2011 [19] Untrained men after acute exercise; randomized trial; n = 18	MSM 50 mg/kg/day; 10 days	Placebo; 10 days	Reduced selected oxidative-stress markers and increased GSH/GSSG ratio; precise between-group estimates were incompletely reported	MSM monotherapy	Very small, model-specific study relying on surrogate outcomes
Withee et al., 2017 [20] Half-marathon participants; double-blind RCT; n = 22	MSM 3 g/day; 23 days	Placebo; 23 days	No significant 8-OHdG, MDA, CK, or LDH effects; muscle-pain difference was not statistically significant (*p* = 0.063)	MSM monotherapy	Underpowered; clinically suggestive pain magnitude did not establish efficacy
Gumina et al., 2012 [37] Repair of large posterosuperior rotator cuff tears; prospective randomized study; n = 90	Tenosan (arginine L-alpha-ketoglutarate + MSM + collagen I + bromelain); 3 months	No supplement; outcomes through 6 months	Lower pain at 6 months and a modest MRI repair-integrity signal; objective function did not significantly differ	MSM-containing combination	No MSM-only arm; component attribution is impossible
Merolla et al., 2015 [38] Arthroscopic supraspinatus repair; randomized placebo-controlled study; 104 randomized, 100 analyzed	Tendisulfur multi-ingredient formulation; 2 months	Placebo + standard analgesia; 24 weeks	Overall pain lower at week 1 (*p* = 0.0477); week 2 *p* = 0.0988; later differences not significant	MSM-containing combination	Early, transient signal; no MSM-only arm
Vitali et al., 2019 [36] Rotator cuff, lateral elbow, or Achilles tendinopathy; comparative study; n = 90	ESWT + Tendisulfur Forte	ESWT alone; 60 days	Better short-term pain and condition-specific scores; UCLA shoulder score *p* = 0.0002 at 60 days	MSM-containing combination	Nonrandomized mixed-condition study; treatment and component confounding
Yoon et al., 2026 [4] Large multilobulated shoulder calcific tendinitis; case report; n = 1	MSM 2 g/day + L-arginine, vitamins, minerals, US-guided prolotherapy, hydration, PT, and activity modification	No comparator; 2 weeks	VAS 10 to 2 and near-complete radiographic resorption	Multimodal intervention	Spontaneous resorption and simultaneous interventions prohibit attribution to MSM

Abbreviations: 8-OHdG, 8-hydroxy-2′-deoxyguanosine; CI, confidence interval; CK, creatine kinase; ESWT, extracorporeal shockwave therapy; GSH/GSSG, reduced-to-oxidized glutathione ratio; JKOM, Japanese Knee Osteoarthritis Measure; KOOS, Knee injury and Osteoarthritis Outcome Score; LDH, lactate dehydrogenase; MDA, malondialdehyde; MRI, magnetic resonance imaging; MSM, methylsulfonylmethane; OA, osteoarthritis; POMS, Profile of Mood States; PT, physical therapy; RCT, randomized controlled trial; US, ultrasound; VAS, visual analog scale; WOMAC, Western Ontario and McMaster Universities Osteoarthritis Index.

**Table 3:** Evidence status and suggested outcome monitoring by clinical domain.

Domain	Current Evidence Level	Potential Role Based on Current Evidence	Appropriate Outcome/Research Monitoring	Key Caution
Knee OA/joint symptoms	Limited short-term human RCT evidence; comparatively most developed MSM indication	Time-limited symptomatic adjunctive trial may be considered in selected patients	Pain + WOMAC/KOOS/JKOM; function; adverse effects	No established structural or disease-modifying effect
Tendinopathy/tendon repair	Formulation-level studies; MSM-specific effect remains unproven	Adjunctive use remains formulation-specific; evidence does not establish an MSM-specific tendon effect	VAS/NRS + VISA-A/UCLA/Constant or imaging when clinically indicated	No MSM-only arm in key tendon studies; component attribution is not possible
Heavy-metal intoxication	No direct human evidence for MSM chelation	No established clinical role for MSM; research hypothesis only	Validated metal-specific blood/urine biomarkers in toxicology research	Do not substitute for DMSA/DMPS/EDTA/other indicated chelation or sodium thiosulfate
Cardiometabolic disease	Small human biomarker trial + preclinical evidence	No established therapeutic role; research context only	Lipids, hs-CRP, glucose/HbA1c, and disease-specific endpoints	No evidence for cardiovascular event reduction
GI/microbiome	Mechanistic/preclinical; little direct human treatment evidence	No established therapeutic role; research context only	Validated GI outcomes in prospective studies	Microbial association does not prove clinical benefit
Exercise recovery/performance	Small, heterogeneous human studies with inconsistent biomarker and symptom findings	No established performance-enhancing or injury-preventive role	Prespecified validated recovery outcomes plus standardized biomarker timing in controlled studies	Model, dose, timing, and endpoint heterogeneity; studies are underpowered

Abbreviations: HbA1c, glycated hemoglobin; hs-CRP, high-sensitivity C-reactive protein; JKOM, Japanese Knee Osteoarthritis Measure; KOOS, Knee injury and Osteoarthritis Outcome Score; MSM, methylsulfonylmethane; NRS, numeric rating scale; OA, osteoarthritis; RCT, randomized controlled trial; VAS, visual analog scale; VISA-A, Victorian Institute of Sport Assessment–Achilles; WOMAC, Western Ontario and McMaster Universities Osteoarthritis Index.

## Data Availability

No new data were created or analyzed in this study. Data sharing is not applicable to this article.

## Supplementary Materials

The following supporting information can be downloaded at: https://www.mdpi.com/article/10.3390/ijms27208967/s1.
