# Supplementary material for: Methylsulfonylmethane as a Bioactive Nutraceutical: Molecular Mechanisms, Clinical Evidence, Combination Therapy, Dosing, and Safety
Source: Int J Mol Sci. 2026 Oct 9;27(20):8967. doi: 10.3390/ijms27208967 (PMC13704369; doi:10.3390/ijms27208967)
Supplement: Supplementary file 1 [file ijms-27-08967-s001.zip › ijms-4593079-supplementary.pdf]

Supplementary Material

Methylsulfonylmethane as a Bioactive Nutraceutical: Molecular Mechanisms, Clinical Evidence, Combination Therapy, Dosing, and Safety

Table S1. Illustrative literature-search framework and evidence-selection approach

This supplement documents the targeted search framework used to inform the narrative review. It is provided to improve transparency and reproducibility without retrospectively recasting the article as a systematic review. Search-result counts were not prospectively logged and are therefore not reconstructed.

Source	Coverage	Search implementation	Role in the review
PubMed/MEDLINE	Database inception to 26 September 2026	MSM synonyms combined with indication, mechanism, formulation, safety, toxicology, and interaction concepts. The reproducible PubMed query is shown below.	Primary identification and verification of peer-reviewed biomedical evidence.
ScienceDirect	Database inception to 26 September 2026	Phrase and synonym searches using “methylsulfonylmethane,” “methyl sulfonyl methane,” and “dimethyl sulfone,” combined with the domain terms listed below.	Supplementary retrieval of primary and review literature and full-text verification when available.
Google Scholar	Database inception to 26 September 2026	Exact-title, exact-phrase, author, formulation, and citation searches; used for supplementary retrieval and forward/backward citation tracing.	Gap filling and source verification; not used to claim exhaustive coverage.
Reference lists	Through 26 September 2026	Backward citation screening of included primary studies and relevant reviews; forward citation checks for key clinical studies when clarification was required.	Identification of additional eligible or companion reports.

Reproducible PubMed/MEDLINE query

(("methylsulfonylmethane"[Title/Abstract] OR "methyl sulfonyl methane"[Title/Abstract] OR "methylsulphonylmethane"[Title/Abstract] OR "dimethyl sulfone"[Title/Abstract] OR "dimethyl sulphone"[Title/Abstract] OR (MSM[Title/Abstract] AND (supplement*[Title/Abstract] OR nutraceutical*[Title/Abstract]))) AND (osteoarthritis[Title/Abstract] OR glucosamine[Title/Abstract] OR chondroitin[Title/Abstract] OR tendin*[Title/Abstract] OR "rotator cuff"[Title/Abstract] OR Achilles[Title/Abstract] OR exercise[Title/Abstract] OR recovery[Title/Abstract] OR cardiovascular[Title/Abstract] OR cardiometabolic[Title/Abstract] OR microbiome[Title/Abstract] OR gastrointestin*[Title/Abstract] OR safety[Title/Abstract] OR toxic*[Title/Abstract] OR warfarin[Title/Abstract] OR anticoagul*[Title/Abstract] OR chelat*[Title/Abstract] OR "heavy metal"[Title/Abstract] OR "NF-kappa B"[Title/Abstract] OR NLRP3[Title/Abstract] OR Nrf2[Title/Abstract] OR AMPK[Title/Abstract] OR mTOR[Title/Abstract] OR autophag*[Title/Abstract] OR glutathione[Title/Abstract] OR sulfur[Title/Abstract]))

Supplementary search concepts

Additional formulation and toxicology terms used in source-specific searches included Tendisulfur, Tenosan, arginine, collagen, xenobiotic, acetaminophen, cisplatin, carbon tetrachloride, paraquat, EDTA, thiosulfate, permeability, pharmacokinetics, adverse events, and drug interaction.

Eligibility criteria

- Peer-reviewed systematic reviews or meta-analyses, randomized or controlled human studies, observational studies, case-level reports, pharmacokinetic or biomarker studies, and safety or interaction reports that specifically evaluated MSM monotherapy or an explicitly identified MSM-containing formulation.
- Animal and in vitro studies retained only for mechanistic questions or clinical domains with sparse or absent human evidence.
- Reports required identifiable MSM exposure or an explicitly described MSM-containing formulation.

Exclusion criteria:

- Non-peer-reviewed promotional material.
- Reports in which MSM exposure could not be verified.
- Nonhuman studies interpreted as direct evidence of clinical efficacy.

- Duplicate or less complete reports when a more complete source described the same study; companion reports were used only to clarify methods or outcomes.

Study selection and narrative synthesis

- Potentially relevant publications were screened for topical relevance at the title/abstract level and assessed in full text when eligibility or interpretation was uncertain.
- The included evidence and its interpretation were reviewed by the authors during manuscript verification.
- Narrative emphasis followed a descriptive hierarchy: systematic reviews or meta-analyses of controlled human studies; randomized controlled trials; nonrandomized or observational human studies; case-level evidence; animal studies; and in vitro studies.
- The hierarchy was not a formal GRADE or certainty-of-evidence assessment.
- No formal risk-of-bias instrument, de novo meta-analysis, or PRISMA flow diagram was applied. Search-result counts were not retrospectively inferred.
